# Habitat selection by Eurasian lynx (*Lynx lynx*) is primarily driven by avoidance of human activity during day and prey availability during night

**DOI:** 10.1002/ece3.3204

**Published:** 2017-07-06

**Authors:** Marc Filla, Joseph Premier, Nora Magg, Claudia Dupke, Igor Khorozyan, Matthias Waltert, Luděk Bufka, Marco Heurich

**Affiliations:** ^1^ Workgroup on Endangered Species J.F. Blumenbach Institute of Zoology and Anthropology Georg‐August University of Göttingen Göttingen Germany; ^2^ Global Change Ecology University of Bayreuth Bayreuth Germany; ^3^ Forest Research Institute of Baden‐Württemberg Freiburg Germany; ^4^ Department of Biometry and Environmental System Analysis University of Freiburg Freiburg Germany; ^5^ Faculty of Forestry and Wood Sciences Czech University of Life Sciences Prague Czech Republic; ^6^ Department of Research and Nature Protection Šumava National Park Administration Kašperské Hory Czech Republic; ^7^ Chair of Wildlife Ecology and Management Faculty of Environment and Natural Resources University of Freiburg Freiburg Germany; ^8^ Department of Conservation and Research Bavarian Forest National Park Grafenau Germany

**Keywords:** diel patterns, disturbance, habitat choice, human activity

## Abstract

The greatest threat to the protected Eurasian lynx (*Lynx lynx*) in Central Europe is human‐induced mortality. As the availability of lynx prey often peaks in human‐modified areas, lynx have to balance successful prey hunting with the risk of encounters with humans. We hypothesized that lynx minimize this risk by adjusting habitat choices to the phases of the day and over seasons. We predicted that (1) due to avoidance of human‐dominated areas during daytime, lynx range use is higher at nighttime, that (2) prey availability drives lynx habitat selection at night, whereas high cover, terrain inaccessibility, and distance to human infrastructure drive habitat selection during the day, and that (3) habitat selection also differs between seasons, with altitude being a dominant factor in winter. To test these hypotheses, we analyzed telemetry data (GPS, VHF) of 10 lynx in the Bohemian Forest Ecosystem (Germany, Czech Republic) between 2005 and 2013 using generalized additive mixed models and considering various predictor variables. Night ranges exceeded day ranges by more than 10%. At night, lynx selected open habitats, such as meadows, which are associated with high ungulate abundance. By contrast, during the day, lynx selected habitats offering dense understorey cover and rugged terrain away from human infrastructure. In summer, land‐cover type greatly shaped lynx habitats, whereas in winter, lynx selected lower altitudes. We concluded that open habitats need to be considered for more realistic habitat models and contribute to future management and conservation (habitat suitability, carrying capacity) of Eurasian lynx in Central Europe.

## 1 INTRODUCTION

Large carnivores are positioned at the top of food webs (Linnell, Salvatori, & Boitani, 2008), which implies naturally low population numbers, high metabolic rates, and great spatial requirements (Ripple et al., 2014). Large home ranges and protein‐rich diets have led to competition and conflicts with humans over shared resources, such as game and livestock (Baker, Boitani, Harris, Saunders, & White, 2008; Treves & Karanth, 2003). As a result, centuries of intense persecution by humans accompanied by habitat loss and a reduction in prey densities have led to global extinctions, local extirpations, and massive range contractions of large carnivore species all over the world (Breitenmoser et al., 2001; Ripple et al., 2014).

Changes in public attitudes toward large carnivores triggered a favorable shift in conservation decision‐making in the middle of the 20th century (Breitenmoser, 1998; Linnell, Swenson, & Anderson, 2001). Reintroduction initiatives aimed at bringing these species back to former habitats and protection measures aspired to stabilize the remaining or newly established populations (Ripple et al., 2014). Today, all European countries concede some form of legal protection of large carnivore species (Chapron et al., 2014; Linnell et al., 2008). However, the return of large carnivores to their original range, which nowadays mostly consists of cultivated landscapes, is accompanied by countless debates that are fuelled by fears of hunters and farmers of depredation of game species and livestock (Linnell, Broseth, Odden, & Nilsen, 2010; Lüchtrath & Schraml, 2015). Such negative perceptions contrast the hopes of managers for the reduction in ungulate populations in managed forests and other landscapes (Hothorn & Müller, 2010; Müller et al., 2014), which, however, still provokes a lot of controversy (Allen et al., in press).

Europe's protected areas often do not meet the spatial requirements of large carnivores, especially for the long‐term viability of populations (Chapron et al., 2014). Hence, the spatial distribution of Eurasian lynx (*Lynx lynx*) populations in Europe overlaps with human‐modified areas. In order to facilitate coexistence of lynx and humans and for successful management and conservation of lynx, it is essential to understand the ecological needs and habitat requirements of lynx (Boitani & Fuller, 2000; Kolowski & Woolf, 2002; Niedzialkowska et al., 2006; Schadt, Revilla, et al., 2002; Zimmermann & Breitenmoser, 2007). Habitat selection can be considered as a hierarchical process in which animals aim to meet their needs at various spatial scales (Johnson, 1980). On a large‐scale equivalent to first‐ and second‐order habitat selection, Eurasian lynx avoid areas of intensive human land use and opt for various forest types with sufficiently high ungulate densities (Breitenmoser et al., 2001, 2015; Magg et al., 2015; Müller et al., 2014; Niedzialkowska et al., 2006). A limited number of studies have investigated habitat selection by lynx on a finer scale (third‐ or fourth‐order selection) and have mainly described microhabitat characteristics, such as significance of low visibility for resting sites and importance of habitat heterogeneity (stalking cover, good visibility) for kill sites (Belotti et al., 2013; Podgórski, Schmidt, Kowalczyk, & Gulczyńska, 2008). Roe deer (*Capreolus capreolus*), the main prey of lynx in Central Europe (Jędrzejewski, Schmidt, Milkowski, Jędrzejewska, & Okarma, 1993), reach good body condition and high densities in human‐modified landscapes (Abbas et al., 2011; Basille et al., 2009; Hewison et al., 2009) which also applies for Central Europe (Heurich et al., 2015; Gehr et al., in press; Märkel et al., unpublished data). Here, the main causes of lynx mortality, poaching and road accidents (e.g., Kaczensky et al., 2013), are related to humans. Thus, lynx have to select habitats that balance prey availability against the risk of encountering humans. Previous studies have investigated this trade‐off and have shown that large‐scale habitat decisions made by lynx constrain their behavior on a fine scale (Basille et al., 2009, 2013; Bouyer et al., 2015; Bunnefeld, Linnell, Odden, Van Duijn, & Andersen, 2006).

As in other regions, humans also pose the greatest threat to the local population of Eurasian lynx in the Bohemian Forest Ecosystem, a protected landscape comprised of the Bavarian Forest National Park in Germany and the Šumava National Park in the Czech Republic. Both inspections of lynx found dead and opinion surveys of local hunters underline that, apart from occasional collisions with vehicles, poaching represents the main cause of lynx mortality in the Bohemian Forest Ecosystem (Červený, Koubek, & Bufka, 2002; Wölfl et al., 2001) which is also indicated by modeling approaches (Müller et al., 2014; Magg et al., 2015; Heurich et al., unpublished data). This is of special concern as the long‐term viability of the local reintroduced lynx population depends on survival within the human‐modified landscape around the protected areas (Belotti et al., 2015; for more details about the study population, see Wölfl et al., 2001). Simultaneously, roe deer are highly abundant in the human‐modified areas (Dupke et al., 2016;Fig.  S1). Under these circumstances, lynx should use habitats relative to variations in perceived risk (Bonnot et al., 2013). According to predictions of the predation risk allocation hypothesis, animals should allocate more effort to feed in low‐risk situations and more anti‐predator effort in high‐risk situations (Lima & Bednekoff, 1999). It is expected that human disturbance as a potential source of risk decreases from daytime, when human activities are likely to affect animals (Belotti, Heurich, Kreisinger, Šustr, & Bufka, 2012), to nighttime.

In this study, we investigated the habitat choice of Eurasian lynx in a human‐altered landscape during the day and at night. We considered a number of anthropogenic, topographic, and environmental variables. We expected that lynx habitat use differs between day and night and that daytime habitat use is shaped by the need for protection from human activities. By contrast, we expected that lynx habitat selection at night is characterized by the availability of and the need to hunt prey.

As all lynx territories in the study area extend from highly protected areas (national parks) into human‐modified landscapes and safety should be prioritized when human activity is high, we first hypothesized that lynx avoid territory edges and, hence, use smaller spatial areas during daytime. Second, we presumed that, apart from remoteness to human activities, lynx select habitats with dense understorey cover (Table S1, for more information, see Latifi et al., 2016) and low accessibility during the day. By contrast, we predicted that habitat selection by lynx at night is mainly driven by the occurrence of roe deer as the main prey in the Bohemian Forest Ecosystem (Belotti et al., 2015; Mayer, Belotti, Bufka, & Heurich, 2012). At night, roe deer occurrence is shaped by high use and selection of open habitats offering high forage availability, such as meadows (Dupke et al., 2016; Fig. S2). Consequently, we expected lynx to select these habitats for hunting activities at night in order to increase their predation success. Third, we predicted that lynx habitat selection differs between summer and winter. In the study region, ungulates move to lower altitudes in winter (Cagnacci et al., 2011; Heurich et al., 2015), where human densities are higher and animals come closer to human infrastructure. Hence, we assumed a similar pattern for lynx and expected safety factors at daytime to be of even higher relative significance in this season.

So far, differences in habitat selection by Eurasian lynx between the phases of the day and between seasons have been poorly investigated. Therefore, we anticipate that results of this study in conjunction with findings from previous research activities in the Bohemian Forest Ecosystem conducted on different spatial scales (Belotti et al., 2012, 2013; Magg et al., 2015) and with recent studies on other lynx populations (Gehr et al., in press) will contribute to successful management and conservation of Eurasian lynx in the study area and beyond.

## 2 MATERIAL AND METHODS

### 2.1 Study area

The Bohemian Forest Ecosystem along the border between Germany and the Czech Republic covers a forested mountain range and is the largest area of strictly protected forest in Central Europe. It includes the Šumava National Park (690 km², 49°7′0″N, 13°36′0″E) and the adjacent Bavarian Forest National Park (240 km², 49°3′19″N, 13°12′9″E). Human densities are relatively low. They vary between less than 2 people per km² in the core area to about 30 and 70 people per km^2^ in the marginal areas in the Czech Republic and Germany, respectively (Heurich et al., 2015).

Altitude inside the protected area ranges from 600 m a.s.l. to 1,450 m a.s.l. The region receives a mean annual precipitation of 965–1,860 mm and a mean annual air temperature of 3.9–8.6°C (Röder et al., 2010). Snow cover at high altitudes can last up to 8 months per year. The highest forest cover is found inside the core zone where it amounts to more than 90% (Fischer, Winter, Lohberger, Jehl, & Fischer, 2013; Heurich, Beudert, Rall, & Křenová, 2010). Norway spruce (*Picea abies*) dominates vegetation at higher altitudes and is complemented by mountain ash (*Sorbus aucuparia*), while Norway spruce, European beech (*Fagus sylvatica*), and silver fir (*Abies alba*) shape lower altitudes (Cailleret, Heurich, & Bugmann, 2014; Heurich & Neufanger, 2005). In the last decades, infestation of Norway spruce with spruce bark beetles (*Ips typographus*) and wind throws have led to large areas of natural disturbance (hereafter referred to as “disturbance areas”; Fahse & Heurich, 2011).

The Eurasian lynx is the only large carnivore species that inhabits the study area permanently and, moreover, occupies almost its entire range. The most common carnivore is the red fox (*Vulpes vulpes*). Roe deer, red deer (*Cervus elaphus*), and wild boar (*Sus scrofa*) are the abundant species of wild ungulates. Roe deer and red deer represent up to about 80% and 17% of lynx kills, respectively (Belotti et al., 2015).

### 2.2 Telemetry data

This investigation is based on GPS and VHF data obtained from 10 lynx (six males and four females). Three individuals were caught as subadults (up to 2 years old) or juveniles (less than 1 year old); however, all of them reached maturity before the end of their monitoring period (Belotti et al., 2015; Table S2). Animals were captured between 2005 and 2012 in baited walk‐through box traps at kill sites and at well‐known lynx trails. Lynx were immobilized using the so‐called Hellabrunn mixture (400 mg ketamine and 500 mg xylazine; Heurich, 2011; Belotti et al., 2015). Immediately after capture and immobilization, all lynx were equipped with GPS‐GSM collars weighing 300 g (VECTRONIC Aerospace, Berlin, Germany; for a detailed description of this procedure, see Heurich, 2011). Both the Ethics Committee of the Government of Upper Bavaria and the Czech Central Commission for Animal Welfare were involved in preparation of the project and approved handling procedures: Ethical criteria concerning research on wild animals were met (permit number: 55.2–1–54–2532–82–10). Moreover, permits for the capture of wild animals were received from the Czech Ministry of Environment (permit number: 41584/ENV/10–1643/620/10–PP8), the Czech Central Commission for Animal Welfare (permit number: 44048/2008–17210, 44048/2008–10001) and the Government of Lower Bavaria (permit number: 55.1–8621.1–57).

Some individuals were captured multiple times. Collars transmitted lynx locations over a period of 3.5–18 months (Table S2). It was expected that the utilized GPS collars functioned with an accuracy of 4–16 m, depending on the habitat type (Stache, Löttker, & Heurich, 2012). Devices stored various amounts of data points per calendar day, but mainly collected data at midnight (00:00 Central European Time: CET = UTC + 1) and at noon (12:00; Belotti et al., 2015). For one male and one female lynx, we additionally included locations collected by VHF data in the analysis to account for gaps in their corresponding GPS data.

### 2.3 Statistical analysis

#### 2.3.1 Data structure

The underlying dataset contained more than 15,000 locations, with each individual contributing between 271 and 2,289 positions. In order to represent the phases of the day with low and high lynx activity, respectively (Heurich et al., 2014; Podolski, Belotti, Bufka, Reulen, & Heurich, 2013), daytime locations were defined as sites sought by lynx between 10:00 a.m. and 02:00 p.m. and nighttime locations were defined as those gathered after nautical dusk and before nautical dawn. Furthermore, we distinguished between the seasons summer (15 April–14 October) and winter (15 October–14 April), following recommendations of Belotti et al. (2013) based on characteristics of leaf fall and snow cover in the study area which are known to affect lynx hunting success and spatial distribution of the prey species (Belotti et al., 2015; Heurich et al., 2015).

#### 2.3.2 Explanatory variables

A total of 12 predictor variables were used for the analysis of lynx habitat use and selection (Table 1).

**Table 1:** Characteristics of predictor variables used for the analysis of habitat selection by lynx in the Bohemian Forest Ecosystem

Predictor variable	Definition	Resolution (m)	Range^a^
Land cover	Land‐cover type	—	—
Altitude	Altitude	10 × 10	574.1–1,413 m a.s.l.
Sun	Monthly solar radiation	10 × 10	3.6–196.3 kWh/m²
TRI	Terrain ruggedness index	10 × 10	15.1–228.3 m
Slope	Slope	10 × 10	0.2°–48.6°
Aspect	Slope direction	10 × 10	0.1°–360°
dTrails	Distance to hiking/cycling trails	10 × 10	0–2,611 m
dRoads	Distance to major roads	10 × 10	0–6,706 m
dSettlement	Distance to human settlements and larger artificial surfaces	10 × 10	0–6,382 m
dRock	Distance to rock formations	10 × 10	0–4,127 m
Roedeer	Relative density of roe deer	500 × 500	0.0–1.8
Reddeer	Relative density of red deer	500 × 500	0.1–2.9

a Considering presence points only.

**Figure 1:**
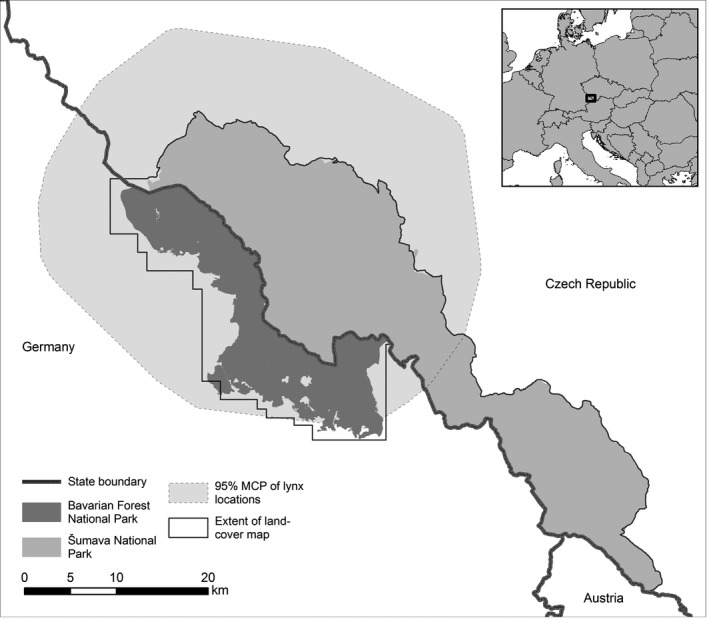
Location of the study area within the Bohemian Forest Ecosystem and Europe (inset), national park boundaries, telemetry locations of lynx, and extent of the utilized land‐cover map. Created in ArcGIS ^®^ 10.3.1

Land‐cover types were assessed with the use of a detailed land‐cover map based on spectrozonal aerial images from 2008 (Suk & Šafár, unpublished report). Consequently, it adequately reflected the habitat structure during the lynx monitoring period (2005–2013). The 26 land‐cover categories encompassed natural vegetation and human‐dominated areas and were merged into nine types based on expert judgments (Table S3). The land‐cover map covered the Bavarian Forest National Park and Šumava National Park almost entirely and in addition comprised some areas in their vicinity; however, it did not include all lynx locations that were available from telemetry (Fig. 1). Hence, we had to reduce the dataset to 3,643 GPS and 321 VHF locations, with each individual contributing between 76 and 704 positions (396 ± 203 *SD*).

Densities of roe deer and red deer were measured to account for the spatial distribution of prey species. For this, we used the standing crop count method on 218 triangular transects in 2010 (see Heurich et al., 2015); this represented a relative measure of ungulate density based on droppings just after snowmelt. Hence, this method was applicable only for the analysis of the winter season.

Altitudinal data were available in the form of a digital terrain model (DTM; Bavarian State Office for Digitizing, Broadband and Surveying; Czech Office for Surveying, Mapping and Cadastre—ČÚZK). Furthermore, monthly solar radiation considering altitude, surface orientation, atmospheric conditions, and topography (Fu & Rich, 2002) was assessed for 2008 and attributed to lynx locations corresponding to the month. Additionally, the elevation model served as a basis for the calculation of slope, aspect, and terrain ruggedness index (TRI; Riley, DeGloria, & Elliot, 1999). The TRI considers the sum of differences in altitudes between a cell and its eight neighboring cells and, hence, provides an objective and quantitative criterion for topographic heterogeneity (Riley et al., 1999).

Distances from features with various degrees of human activity (settlements, roads, and trails) to lynx locations were determined to identify how human infrastructure affected lynx behavior. The administrations of Šumava National Park and Bavarian Forest National Park provided routes of hiking and cycling trails. Distances from lynx locations to major roads (km) were measured using data from OpenStreetMap (http://download.geofabrik.de) for Germany and the Czech Republic. Motorways, trunks, and primary, secondary, tertiary, and unclassified (>200 m in length) roads as well as corresponding link roads were incorporated into this analysis. Smaller and infrequently used minor roads were neglected (for a detailed description of the road categories, see OpenStreetMap Wiki 2016). Moreover, we calculated distances to human settlements located on the Czech side (Czech Office for Surveying, Mapping and Cadastre—ČÚZK) and on the German side (Bavarian State Office for Digitizing, Broadband and Surveying) of the border. We also created a distance variable for rock formations (Bavarian State Office for Digitizing, Broadband and Surveying; Czech Office for Surveying, Mapping and Cadastre—ČÚZK) to explore the importance of this natural habitat, which was expected to be insufficiently covered on the land‐cover map.

All variables were mapped and analyzed in ArcGIS 10.3.1 (ESRI, Redlands, CA, USA).

#### 2.3.3 Range requirements

For the 10 lynx captured and monitored in the study area, we calculated home ranges as 95% minimum convex polygons (MCP) and range uses (Kernel 90) by applying the reference bandwidth as the smoothing factor. The quantity of daytime and nighttime locations was reduced to a maximum of one per calendar day each in order to limit both temporal and spatial autocorrelation of telemetry data. In addition, the time period was restricted to a maximum of 1 year (365 days). Furthermore, we checked for possible home range shifts in this period by plotting 95% MCPs over time (Fig. S3). Differences in spatial range sizes between sexes and phases of the day were assessed by applying the Mann–Whitney *U*‐test and the Wilcoxon signed rank test.

#### 2.3.4 Modeling habitat use and selection

To reduce spatial and temporal autocorrelation, we limited daytime and nighttime datasets to one location per calendar day and individual lynx. Moreover, data points outside 95% MCPs were excluded to account for outliers in individual range use. As we did not want to calculate “exact” home ranges but define areas that were used by lynx during the entire study period, shifts in home ranges were admitted in habitat analysis to allow for concomitant changes in lynx habitat use.

The realized locations provided information about the presence of Eurasian lynx in the study area. We analyzed both daytime and nighttime habitat use and additionally applied Pearson's χ^2^‐test to compare seasonal use. For the study of habitat selection, we compared the selected sites with those that are available in the study area. As recommended by Barbet‐Massin, Jiguet, Albert, and Thuiller (2012), we randomly selected a high number of absence points by drawing randomly 10 times as many random absence points for each individual within its spatial range (95% MCP) and the boundaries of the land‐cover map resulting in about 10,000 points for each of the four time slots.

To investigate factors that impact Eurasian lynx occurrence, we applied generalized additive mixed models. The flexible characteristics of this model type were expected to fit various predictor variables best. In total, four different models were run for the two seasons and two phases of the day, with lynx presence as the dependent variable, various predictor variables (Table 1), and the lynx individual as a random factor to account for differences in sample sizes and individual preferences (Gillies et al., 2006). Correlations of predictor variables were checked using Spearman's correlation. A variable regarded as less important was excluded from a model when predictors had a Spearman correlation coefficient modulus equal to or greater than 0.7, following the threshold proposed by Dormann et al. (2013). Consequently, we excluded the predictors “Slope” and “dRoads” from the analysis, as they highly correlated with “TRI” and “dSettlement,” which were considered to be of higher explanatory value. In addition, the predictor “Aspect” was given preference over the variable “Sun” due to better comparability with similar studies (e.g., Donovan et al., 2011; Husseman et al., 2003). The individual L10 was excluded from winter models as the sample size was too low (*n *= 5 and *n *= 6 for daytime and nighttime locations, respectively).

Relative variable importance for the two phases of the day and two seasons was examined in a permutation procedure. This method is based on randomization of one predictor variable and comparison of correlation coefficients between predictions of the original and the “new randomization‐based” model (Thuiller, Lafourcade, Engler, & Araújo, 2009). Each environmental variable was randomized 10 times. A raw importance value was calculated for each variable as one minus the mean correlation between predicted lynx occurrences using the original and randomized models (Heurich et al., 2015; Thuiller et al., 2009). Importance values were standardized to a sum of one.

Statistical analyses were conducted with the software R Studio version 3.1.2 (R Core Team 2015a). The following R packages were used during various procedures: adehabitatHR (Calenge, 2006), foreign (R Core Team 2015b), lattice (Sarkar, 2008), maptools (Bivand & Lewin‐Koh, 2016), mgcv (Wood, 2011), plyr (Wickham, 2011), rgdal (Bivand, Keitt, & Rowlingson, 2015), sp (Pebesma & Bivand, 2005), and stats (R Core Team 2015a).

## 3 RESULTS

### 3.1 Range requirements

The male lynx L8 shifted his home range markedly (Fig. S3); hence, only a period of 9 months was considered for this individual. A lack of diurnal locations for male L5 prevented us from calculating meaningful range requirements during daytime for this individual.

Both during the day and at night, male lynx had significantly larger spatial area requirements for their home ranges than females (daytime: *W* = 0, *p *= .029; nighttime: *W *= 0, *p *= .016) and also for their kernel areas (daytime: *W *= 0, *p* = .029; nighttime: *W* = 0, *p* = .016). Moreover, spatial ranges calculated from lynx daytime locations were significantly smaller than those obtained from nighttime locations (95% MCP: *V* = 5, *p* = .020; Kernel 90: *V* = 4, *p* = .014). On average, kernel areas and home ranges derived from nighttime locations exceeded those derived from daytime locations by 12 ± *SE* 5% and 11 ± *SE* 5%, respectively (Table 2).

**Table 2:** Annual home ranges (95% MCP) and range uses (Kernel 90) of 10 radio‐collared lynx in the Bohemian Forest Ecosystem, and the change (%) between daytime‐ and nighttime calculations

Individual	Sex	95% MCP			Kernel 90			Period (days)
		Day (km²)	Night (km²)	Change (%)	Day (km²)	Night (km²)	Change (%)	
L1	m	513	559	+9	507	556	+10	365
L2	m	222	253	+14	283	322	+14	365
L3	f	155	142	−8	186	166	−11	365
L4	f	93	101	+9	125	130	+4	365
L5	m	312	550	+77	611	756	+24	365
L6	m	534	556	+4	716	756	+5	365
L7	f	152	164	+8	206	212	+3	365
L8	m	351	350	±0	486	493	+2	272
L9	f	73	104	+41	74	110	+49	365
L10	m	186	252	+35	345	416	+21	94
Mean	m	405^a^	454^b^	+12^c^	498^a^	576^b^	+10^c^	
	f	118	128	+12	148	155	+11	
	All	262^a^	279^b^	+12^c^	323^a^	389^b^	+11^c^	

a Excluding individuals L10 and L5 due to small sample size.

b Excluding L10 due to small sample size.

c Excluding L5 due to imbalance between number of daytime and nighttime locations.

### 3.2 Habitat use

**Figure 2:**
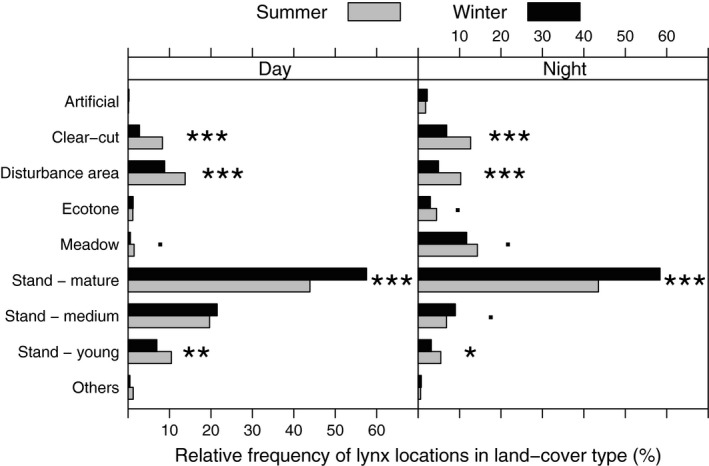
Use of land‐cover types by lynx in the Bohemian Forest during the day and at night. Significance levels of Pearson's χ^2^‐test indicated by 0*** < 0.001** < 0.01* < 0.05 < . < 0.1 indicates the significance of the relationship between the habitat selected and the season. Created in R Studio 3.1.2

The majority of daytime locations, specifically 44% of the summer daytime locations and 58% of the winter daytime locations, were in forest areas with mature stands. In addition, 20% and 21% of lynx daytime locations in summer and winter, respectively, were located in forest composed of medium stands. Disturbance areas (14%), young stands (10%), and clear‐cuts (8%) contributed to a considerable proportion of summer resting sites, but these habitats were less frequently visited in winter (disturbance area: χ^2^ = 10.88, *p* < .001; clear‐cuts: χ^2^ = 25.95, *p* < .001; young stands: χ^2^ = 6.78, *p* = .009). All other habitat categories accounted for less than 5% of daytime habitat use (Fig. 2). Of the lynx daytime locations in winter and summer, 43% and 40%, respectively, were in coniferous forests (Table S4). Mixed forests were more frequently visited in winter (29%) than in summer (20%; χ^2^ = 17.78, *p* < .001). Of the daytime locations in both seasons, 14% were in deciduous forests (Table S4).

Mature stands were the most used habitat type at night and of higher importance in winter than in summer (χ^2^ = 44.54, *p* < .001). In both seasons, meadows made up the second highest proportion of nighttime locations. Clear‐cuts and disturbance areas were often used during summer nights, but they were less frequently used than medium stands on winter nights (Fig. 2). About a third of all lynx positions at nighttime were located in coniferous forest (31% in summer, 32% in winter; Table S4). Deciduous stands were more frequently used on winter nights (13%) than on summer nights (8%; χ^2^ = 12.82; *p* < .001), the same was true for mixed stands, which accounted for 26% and 17% of nighttime locations in winter and summer, respectively (χ^2^ = 27.40, *p* < .001; Table S4).

### 3.3 Habitat selection

**Figure 3:**
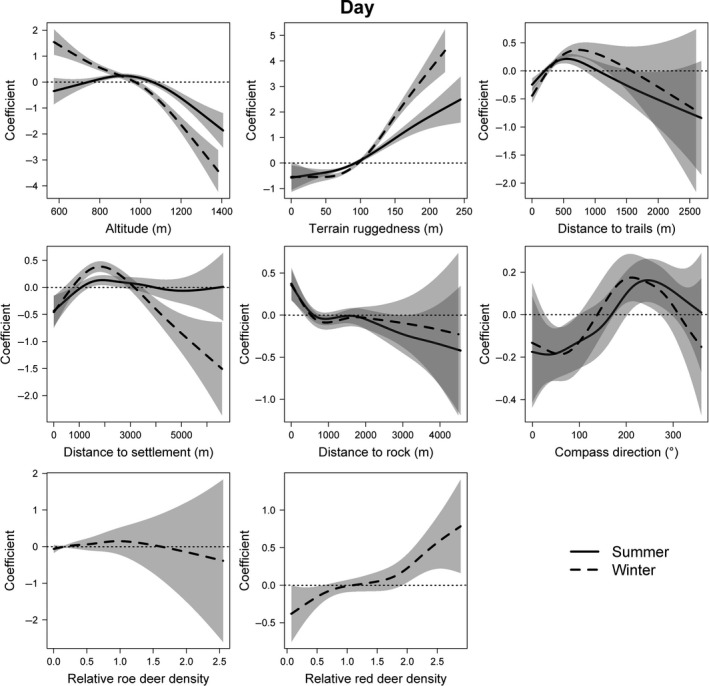
Plots of generalized additive mixed models predicting habitat selection by lynx in the Bohemian Forest Ecosystem during daytime. Created in R Studio 3.1.2

Lynx selected disturbance areas, medium stands, and young stands over mature stands (Intercept of the models) during the day in both seasons, and they selected clear‐cuts and other natural habitats in summer (Table 3). Meadows were avoided during daytime in both summer and winter. With regard to tree species, lynx slightly preferred deciduous forests over coniferous forest in summer (Table S5). In addition, altitude, terrain ruggedness, and aspect as well as distances to rocks, trails, and settlements had a significant influence on daytime habitat selection. The same also applied to red deer densities and lynx individuality (random effect) in winter (Table 3). Lynx selected resting sites within a few hundred meters of rock formations, avoided trails at a similar distance and kept far larger distances to human settlements in summer (Fig. 3). During daytime, lynx locations were in more rugged terrain and on slopes facing southwest or west. Medium altitudes were selected on summer days, whereas in winter, animals selected lower altitudes and avoided regions located higher than 1,000 m a.s.l. Areas with very high red deer density were selected on winter days (Fig. 3). The models explained 13.4% and 23.1% of the deviance in summer (*n* = 10,771, adjusted *R* ^2^ = .107) and winter (*n* = 9,810, adjusted *R* ^2^ = .192), respectively.

**Table 3:** Summary of generalized additive mixed models predicting habitat selection by lynx in the Bohemian Forest Ecosystem during daytime. The estimates of the coefficients, standard errors (*SE*), *z* values, and *p*‐values (=Pr(>|*z*|)) are shown for land‐cover types, and the estimated degrees of freedom (edf), residual degrees of freedom (Ref.df), chi‐square test statistics (χ^2^), and *p*‐values (*p*) refer to the summary statistics of the estimation of the spline functions for the continuous explanatory variables

Variables	Summer				Winter			
	Estimate	*SE*	*z* value	Pr(>|*z*|)	Estimate	*SE*	*z* value	Pr(>|*z*|)
Parametric coefficients								
(Intercept)	−2.952	0.058	−51.132	<.001	−3.069	0.12	−25.618	<.001
Artificial	−1.469	1.01	−1.455	.146	−1.066	0.722	−1.475	.14
Clear‐cut	1.424	0.141	10.083	<.001	0.367	0.225	1.633	.102
Disturbance area	1.504	0.133	11.273	<.001	1.664	0.17	9.783	<.001
Ecotone	0.171	0.319	0.535	.592	0.477	0.334	1.431	.153
Meadow	−1.054	0.277	−3.799	<.001	−1.864	0.456	−4.091	<.001
Others	1.249	0.329	3.795	<.001	−0.29	0.553	−0.525	.6
Stand‐medium	1.179	0.097	12.151	<.001	1.267	0.101	12.533	<.001
Stand‐young	2.359	0.143	16.542	<.001	2.275	0.178	12.751	<.001

	edf	Ref.df	χ^2^	*p*	edf	Ref.df	χ^2^	*p*
Approximate significance of smooth terms								
s(Altitude)	2.661	3.393	54.09	<.001	2.334	3.018	145.24	<.001
s(TRI)	2.311	2.983	114.40	<.001	2.569	3.292	328.70	<.001
s(Aspect)	3.339	4.152	14.081	.008	3.202	3.988	13.62	.01
s(dTrail)	2.119	2.675	41.072	<.001	1.896	2.402	59.615	<.001
s(dSettlement)	2.808	3.536	11.037	.012	2.241	2.863	72.594	<.001
s(dRock)	2.652	3.326	11.764	.006	2.473	3.119	12.585	.004
s(Roedeer)	—	—	—	—	1.849	2.368	1.003	.655
s(Reddeer)	—	—	—	—	2.665	3.369	12.642	.008
s(Individual)	0.69	9	0.852	.267	6.58	8	40.676	<.001

On both summer and winter nights, lynx selected meadows, clear‐cuts, young stands, ecotones, and artificial surfaces over mature stands (Intercept of the models). The same was true for disturbance areas on summer nights (Table 4). On winter nights, lynx selected forests composed of deciduous and mixed stands over coniferous stands (Table S5).

**Table 4:** Summary of generalized additive mixed models predicting habitat selection by lynx in the Bohemian Forest Ecosystem during nighttime. The estimates of the coefficients, standard errors (*SE*), *z* values, and *p*‐values (=Pr(>|*z*|)) are shown for land‐cover types, and the estimated degrees of freedom (edf), residual degrees of freedom (Ref.df), chi‐square test statistics (χ^2^), and *p*‐values (*p*) refer to the summary statistics of the estimation of the spline functions for the continuous explanatory variables

Variables	Summer				Winter			
	Estimate	*SE*	*z* Value	Pr(>|*z*|)	Estimate	*SE*	*z* Value	Pr(>|*z*|)
Parametric coefficients								
(Intercept)	−2.715	0.047	−57.283	<.001	−2.683	0.091	−29.481	<.001
Artificial	0.798	0.243	3.28	.001	0.699	0.26	2.692	.007
Clear‐cut	1.617	0.109	14.876	<.001	0.997	0.153	6.504	<.001
Disturbance area	0.872	0.126	6.914	<.001	0.266	0.178	1.495	.135
Ecotone	1.229	0.166	7.405	<.001	0.74	0.223	3.317	.001
Meadow	0.809	0.1	8.087	<.001	0.465	0.122	3.822	<.001
Others	−0.255	0.399	−0.64	.522	0.216	0.414	0.523	.601
Stand‐medium	0.02	0.127	0.155	.877	0.119	0.126	0.942	.346
Stand‐young	1.289	0.151	8.558	<.001	0.604	0.21	2.881	.004

	edf	Ref.df	χ^2^	*p*	edf	Ref.df	χ^2^	*p*
Approximate significance of smooth terms								
s(Altitude)	2.713	3.469	7.382	.116	2.117	2.731	142.78	<.001
s(TRI)	2.152	2.783	2.717	.434	2.15	2.765	61.005	<.001
s(Aspect)	3.596	4.46	3.932	.476	3.326	4.139	31.605	<.001
s(dTrail)	2.323	2.913	23.004	<.001	2.204	2.775	4.618	.16
s(dSettlement)	3.103	3.887	11.729	.018	2.695	3.396	6.826	.118
s(dRock)	2.807	3.532	8.446	.042	2.487	3.149	13.272	.005
s(Roedeer)	—	—	—	—	2.02	2.592	5.32	.216
s(Reddeer)	—	—	—	—	2.731	3.447	13.732	.005
s(Individual)	0.008	9	0.004	.89	5.941	8	26.093	<.001

**Figure 4:**
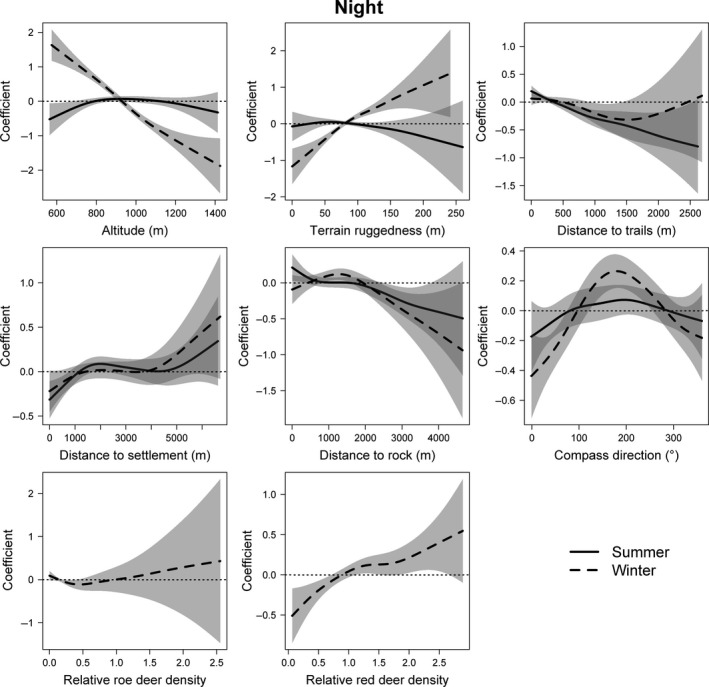
Plots of generalized additive mixed models predicting habitat selection by lynx in the Bohemian Forest Ecosystem during nighttime. Created in R Studio 3.1.2

Distances to human infrastructure (trails, settlements) and rock formations were significant environmental predictors for lynx occurrence on summer nights. This was true for the individual, terrain ruggedness, altitude, aspect, distances to rocks, and red deer densities in winter (Table 4). Lynx especially favored lower altitudes (below 900 m a.s.l.) on winter nights, but avoided very low altitudes on summer nights (Fig. 4). Moreover, areas in close proximity to rock formations and trails were selected on summer nights, whereas settlements were avoided within a radius of 1,000 m on summer nights. On winter nights, lynx selected rugged terrain with south‐ or west‐facing slopes. Lynx selected regions with higher red deer densities on winter nights, whereas roe deer densities had no clear effect in our models (Fig. 4). Deviance explained by the generalized additive mixed models accounted for less than 10% on both summer (*n* = 12,793, adjusted *R* ^2^ = .037) and winter nights (*n* = 10,053, adjusted *R* ^2^ = .044).

### 3.4 Variable importance

The relative importance of predictor variables differed between the phases of the day and between seasons.

In summer, land cover and, to a lower extent, terrain ruggedness were the most important explanatory variables for habitat selection by lynx during daytime. By contrast, the relative importance of terrain ruggedness as the most significant predictor exceeded that of land cover and altitude on winter days. In daylight hours, these three variables contributed to about 90% of the standardized variable importance (Table 5).

**Table 5:** Variable importance (%) in the final selected generalized additive mixed models. A raw importance value was calculated for each variable as one minus the mean correlation between predicted lynx occurrences using the original and randomized models (see details in the text)

Variable	Day		Night	
	Summer	Winter	Summer	Winter
Land cover	62.5	27.4	83.8	13.7
Altitude	8.9	21.6	1.6	49.2
TRI	21.0	35.8	0.7	15.2
Aspect	1.7	0.9	1.0	6.4
dTrails	2.5	3.4	7.3	1.0
dSettlement	1.4	4.8	3.0	1.5
dRock	1.9	0.9	2.6	2.4
Roedeer	—	0.1	—	0.7
Reddeer	—	1.4	—	4.6
Individual	0.0	3.6	0.0	5.2

On summer nights, land cover was the dominant variable affecting lynx habitat choice, whereas altitude was most important on winter nights, followed by terrain ruggedness and land cover (Table 5).

## 4 DISCUSSION

The analysis of telemetry data in the Bohemian Forest Ecosystem yielded new information about the habitat choice of Eurasian lynx on a home‐range scale (third‐order habitat selection). The results revealed a clear difference in habitat preferences between daytime and nighttime. In accordance with our predictions, at night lynx used larger areas and selected open habitats that are associated with high prey abundance. In line with previous studies (e.g., Bouyer et al., 2015), daytime resting sites were in areas providing dense understorey cover and rugged terrain, remote from human infrastructure. As expected, habitat selection also differed between seasons, with habitat choices being strongly influenced by land‐cover types in summer and by altitude in winter.

In general, our GAMMs were able to explain a relatively low proportion of the deviance, which is not uncommon in ecological models (i.e., May et al., 2008; Warren, Wallin, Beausoleil, & Warheit, 2016). However, this indicates that other factors not included in the models may be relevant to the habitat selection by Eurasian lynx. For instance, more precise data on vegetation characteristics (e.g., vegetation density) and human disturbance (e.g., visitor activity in the protected areas), which were available only for parts of the study area, may have led to better model performances. A bias may have been introduced into our results of habitat selection, as we could only use about two‐thirds of all lynx telemetry locations and as environmental and anthropogenic variables might differ between national parks and less‐protected lands in the surroundings (Heurich et al., 2015). Despite full legal protection of lynx in Germany and the Czech Republic, the distance to protected areas is the main factor shaping the distribution of the Eurasian lynx population in the Bohemian Forest Ecosystem (Müller et al., 2014), and killing by humans is the main cause of mortality (e.g., Červený et al., 2002; Wölfl et al., 2001). Thus, lynx are better protected from illegal killing inside the Bavarian Forest National Park and Šumava National Park and differences in habitat selection between day and night might be even more distinct beyond these protected areas. However, areas outside the national parks and villages inside the national park boundaries, particularly on the Czech side, contribute to a gradient of human density in the study area. Additionally, during the day, lynx are also influenced by recreational activities inside the core areas of the parks (Belotti et al., 2012). Finally, all lynx monitored in this study occupy territories that cover parts of the national parks and adjacent unprotected landscapes. Thus, the experience of individual lynx in either of the two areas will affect habitat selection in the other. Consequently, we conclude that this data restriction does not alter the main results, but might possibly mitigate differences between daytime and nighttime habitat selection because of a reduced risk of persecution within the national parks.

In line with our second prediction, a major finding of our study is the use and selection of open habitats by lynx at night, with meadows being the second most frequently used land‐cover type. In the study area, roe deer represent the main prey of lynx and commonly use meadows for foraging between dusk and dawn, especially in summer (Dupke et al., 2016; Fig. S2). However, the presence of ungulate prey alone does not necessarily make a habitat a successful hunting area. A typical hunting strategy of most felids begins with a crouching approach and ends with a short attack (Sunquist & Sunquist, 2002), and long‐distance chasing is quite rare, particularly for lynx (Krofel, Potočnik, & Kos, 2007). Hence, lynx require sufficient cover to get close to prey targets. The significance of habitat complexity and heterogeneity in visibility at kill sites of lynx and other felids is highlighted in numerous studies (Balme, Hunter, & Slotow, 2007; Belotti et al., 2013; Holmes & Laundré, 2006; Podgórski et al., 2008). It is therefore not surprising that lynx in the Bohemian Forest Ecosystem also selected those land‐cover types at night that offered good opportunities for the whole predatory behavioral sequence including detection, ambush and attacking, that is, clear‐cuts and ecotones. Regarding the selection of meadows, it could be assumed that high grasses provide good cover for lynx as they stalk their prey, and hence, serve the same purpose as tree stumps, regenerating forest or coarse woody debris in clear‐cuts. Similarly, Rolley and Warde (1985) found that grassy, bushy areas are increasingly used by bobcats (*Lynx rufus*) in the late afternoon and at night and argued that the bobcats use clear‐cuts and forest openings because of high prey densities. In accordance with these findings, Poole, Wakelyn, and Nicklen (1996) reported that Canada lynx (*Lynx canadensis*) select meadows (second‐order habitat selection) but hardly use them during the day; the authors suggested that open areas might be used at dusk, dawn, and night. So far, open and deforested habitats have received little attention in habitat modeling of Eurasian lynx and have mainly been associated with avoidance by lynx (Niedzialkowska et al., 2006; Schadt, Knauer, et al., 2002). However, our study showed that such open areas are readily used by lynx at night when human activity is low and open areas offer high availability of prey.

In contrast to our expectations, according to our models, large‐scale differences in prey densities had only a low relative significance for nighttime habitat selection. This is probably due to the coarseness of the prey density data, which could not represent the fine‐scale differences in habitat use by lynx. Lynx are efficient hunters even when roe deer densities are very low, for example, about 2 individuals per km^2^ in a Norwegian study (Nilsen, Linnell, Odden, & Andersen, 2009). This is likely to be the case for most parts of the Bavarian Forest National Park, which has an estimated roe deer density of 1–5 individuals per km² (Heurich et al., 2012). Densities of red deer are slightly lower (Heurich et al., 2012). However, this species is managed in both national parks and up to 80% and 50% of the red deer population on the German and Czech side, respectively, spend the winter in enclosures (Heurich, Baierl, Günther, & Sinner, 2011; Heurich et al., 2015). Enclosures were disregarded in the pellet count (Heurich et al., 2015) that provided prey density data utilized in this study. Consequently, variables reflecting prey availability, particularly of roe deer, might influence lynx hunting activities in the Bohemian Forest on a finer scale than our data reflected.

In line with our second prediction, we found that lynx select day sites located in dense habitats characterized by inaccessibility and remoteness. During the day, lynx avoided potentially dangerous open habitats (Lone et al., 2014), for example, meadows (Fig. S1), but selected a variety of land‐cover types over the most common mature forest, such as young to medium stands, disturbance areas, and clear‐cuts. These land‐cover types share similar features in that they provide dense shelter in the form of strong undergrowth, root plates, or fallen logs, which provide good cover (Table S1). Such habitats allow lynx to rest and sleep (Heurich et al., 2014) without facing the danger of being spotted and thereby minimize the risk of encounters with humans. Similarly, Canada lynx select regenerating forest with a dense understorey over mature forests (Mowat & Slough, 2003) and day sites of another close relative, the Iberian lynx (*Lynx pardinus*), are located in similar habitats with tall shrub cover and dense overall understorey (Palomares, 2001). The importance of young thickets and dense undergrowth for resting Eurasian lynx has also been stressed (e.g., Podgórski et al., 2008). Accordingly, Sunde, Stener, and Kvam (1998) found a positive correlation between vegetation cover and the tolerance of resting lynx to humans.

In addition to dense habitats, the lynx tracked in this study strongly selected rugged terrain for the daytime resting sites, which is consistent with results of previous studies of Eurasian lynx (Bouyer et al., 2015; Sunde et al., 1998). Steepness and a high variability in terrain reduce the probability that humans use and visit an area (Basille, Calenge, Marboutin, Andersen, & Gaillard, 2008; Bouyer et al., 2015), which might explain lynx behavior at times of particular vulnerability. During the day, lynx also stayed away from human infrastructure by avoiding trails by up to 300 m and settlements by up to about 1,000 m. This is in line with previous studies that focused on tolerance of carnivores towards human installations. As an example, pumas (*Puma concolor*) maintain larger distances to houses than to arterial roads (Wilmers et al., 2013). Similar to our study, Sunde et al. (1998) have found that Eurasian lynx in Norway avoid houses and roads by 200 m. Belotti et al. (2012) have shown that lynx move longer distances between rest sites and kill sites if a prey carcass is cached in areas with high recreational activities. Our findings are in accordance with lynx studies at larger scales which illustrate the avoidance of areas characterized by intense human land use (Magg et al., 2015; Niedzialkowska et al., 2006). In agreement with Sunde et al. (1998) who suggested that diurnal avoidance of artificial areas is based on human presence rather than on habitat alteration, lynx hardly used trails and roads during daytime but selected them at night. Similar differences in habitat use have also been observed for other predators, such as pumas and leopard cats (*Prionailurus bengalensis*; e.g., Dickson, Jenness, & Beier, 2005; Mohamed et al., 2013). Adding to the latter and in accordance with our first hypothesis, we found lynx to also use larger spatial areas at nighttime than during daytime. To our knowledge, no other study on Eurasian lynx has investigated differences in home range sizes by considering locations during various phases of the day. In the study area, human population density increases from core zones of the national parks to adjacent areas and surrounding landscapes (Heurich et al., 2015). The territories of all lynx that were included in this analysis extend to these less protected areas outside the national parks. Hence, animals that move along some of their territory borders are more likely to encounter humans and expose themselves to a greater risk (Müller et al., 2014) which may explain why these areas are increasingly used at nighttime when human activity is low. However, the use of territory borders during nighttime may also be explained by other factors, such as interactions with conspecifics or scent‐marking behavior when lynx are active (Vogt, Zimmermann, Kölliker, & Breitenmoser, 2014).

Interestingly, lynx selected day sites close to rock formations. The significance of this natural feature for resting animals of Eurasian lynx populations has hardly been addressed. Rock formations provide cover for resting felids (Kolowski & Woolf, 2002), offer good vantage points for prey spotting, provide cover for stalking, and enable lynx to approach prey silently (Krofel et al., 2007). These factors may explain the observed preference of rock formations also on summer nights.

In accordance with our third hypothesis, lynx selected habitat differently between summer and winter both during the day and at night. Land‐cover type was by far the most important predictor for lynx habitat selection in summer but had a lower significance in winter. By contrast, the relative importance of altitude increased from summer to winter. These results are driven mainly by movement patterns of ungulate populations in the Bohemian Forest Ecosystem. Roe deer are relatively uniformly distributed in summer, but avoid harsh winter conditions at higher altitudes by migrating to lower altitudes (Cagnacci et al., 2011; Heurich et al., 2015). To maintain their food supply, lynx have to follow their main prey. Consequently, altitude is the main driver of habitat selection by lynx in winter at night. Lynx movements to lower altitudes and areas outside the national parks in winter involve higher probabilities of encounters with hunters, which might lead to higher mortality (Červený et al., 2002; Magg et al., 2015; Müller et al., 2014; Wölfl et al., 2001). Thus, it seems consistent that day habitat selection in winter is driven even more by safety factors, such as terrain inaccessibility due to high ruggedness, than in summer. This is in accordance with a remark of Bouyer et al. (2015) based on studies of Basille et al. (2008, 2009), that the strength of preference given to rugged terrain increases with the degree of human landscape modification. By contrast, the summer distribution of roe deer allows lynx to hunt in all parts of the Bohemian Forest Ecosystem, including core areas of the national parks, which are less intensely disturbed by humans (Belotti et al., 2015). Therefore, and because the various natural vegetation zones provide different degrees of foraging success, nighttime habitat selection in summer is mainly shaped by land‐cover type. During both daytime and nighttime, forests composed of mature stands were used more frequently in winter than in summer. By contrast, the use of clear‐cuts, disturbance areas, and young stands decreased from summer to winter. This pattern can again be explained by roe deer behavior. In the Bavarian Forest National Park, as temperature decreases and snow depth increases, these ungulates prefer high canopy cover (Ewald, Dupke, Heurich, Müller, & Reineking, 2014). Selection of mature forests with high canopy cover under extreme environmental conditions has also been documented for other ungulate species (Armleder, Waterhouse, Keisker, & Dawson, 1994; van Beest, Van Moorter, & Milner, 2012). As for ungulates, mature forests might also serve as thermal shelters for resting lynx, which would result in a higher use of this forest type on winter days than in summer. The higher use of these habitats by lynx on winter nights can probably be attributed to improved possibilities of hunting ungulate prey.

## 5 CONCLUSION

This study clearly demonstrated a modification of third‐order habitat selection by lynx between daytime and nighttime as well as between summer and winter, which revealed behavioral mechanisms that allow lynx to adapt to human‐modified landscapes. Habitat selection during daytime is mainly driven by safety factors, whereas prey availability determines lynx habitat selection at night. In winter, lynx are forced to take higher risks as ungulates migrate to areas closer to human settlements.

In contrast to forested areas, the significance of open habitats, such as meadows, has rarely been addressed in lynx research. Our analysis emphasizes that open land‐cover types form an important natural habitat for Eurasian lynx at night and should be considered when analyzing landscapes in regard to habitat suitability or carrying capacity for this predator. Overall, the understanding achieved in this study provides new insights into the habitat choice of Eurasian lynx, which can be used to improve conservation and management of this protected felid in Central Europe.

## AUTHOR CONTRIBUTIONS

Marc Filla performed data analysis and interpretation, drafting the article, final approval of the version to be published. Joseph Premier performed data analysis and interpretation, critical revision of the article, final approval of the version to be published. Nora Magg performed data analysis, critical revision of the article, final approval of the version to be published. Igor Khorozyan and Matthias Waltert performed data interpretation, critical revision of the article, final approval of the version to be published. Luděk Bufka performed data collection, critical revision of the article, final approval of the version to be published. Marco Heurich performed design of the work, data collection, data analysis and interpretation, critical revision of the article, final approval of the version to be published.

## CONFLICT OF INTEREST

The authors declare that they have no conflict of interest.

## DATA AVAILABILITY STATEMENT

Datasets analyzed in this study are available from M. Heurich (marco.heurich@npv-bw.bayern.de) upon reasonable request.
