# Supplementary material for: Habitat selection by Eurasian lynx (Lynx lynx) is primarily driven by avoidance of human activity during day and prey availability during night
Source: Ecol Evol. 2017 Jul 6;7(16):6367–81. doi: 10.1002/ece3.3204 (PMC5574813; doi:10.1002/ece3.3204)
Supplement: Supplementary file 4 [file ECE3-7-6367-s004.docx]

**Table S1** Vegetation cover (mean values and standard deviation) for the land-cover types used in this study at different heights (canopy (2-60m), shrub (0.5-5m), understorey (0.5-2m)). Vegetation cover was derived from airborne LiDAR data acquired using a Riegl 680i laser scanner (350 KHz, nominal point density 30-40 points/m^2^; average altitude 650 m) at a 0.32 m footprint. Data were acquired under leaf-on conditions within three days in June 2012 from the entire Bavarian Forest National Park area. LiDAR measurement points were grouped into a 5 × 5 m grids and for each grid cell, inverse penetration ratios were calculated at different heights (2-60 m, 0.5-5 m, 0.5-2 m) above ground. The penetration ratio is an estimation of the fractional vegetation cover at this height (for more information, see Ewald et al. 2014, Latifi et al. 2016).

|  | canopy | canopy_sd | shrub | shrub_sd | understorey | understorey_sd |  |  |
| --- | --- | --- | --- | --- | --- | --- | --- | --- |
| Artificial | 0.36433 | 0.32718 | 0.30504 | 0.27356 | 0.08082 | 0.10871 |  |  |
| Clear-cut | 0.25475 | 0.24344 | 0.28047 | 0.24470 | 0.10668 | 0.12147 |  |  |
| Disturbance area | 0.31453 | 0.24796 | 0.34283 | 0.21407 | 0.18857 | 0.15738 |  |  |
| Ecotone | 0.35965 | 0.32564 | 0.29317 | 0.26458 | 0.09893 | 0.12887 |  |  |
| Meadow | 0.18673 | 0.21241 | 0.23172 | 0.24893 | 0.02564 | 0.06099 |  |  |
| Others | 0.27201 | 0.12504 | 0.57050 | 0.11740 | 0.33073 | 0.08241 |  |  |
| Stand - mature | 0.70648 | 0.34241 | 0.25488 | 0.22502 | 0.09778 | 0.13699 |  |  |
| Stand - medium | 0.66995 | 0.33533 | 0.38116 | 0.28224 | 0.16773 | 0.18437 |  |  |
| Stand - young | 0.36307 | 0.27242 | 0.44462 | 0.29761 | 0.26496 | 0.22186 |  |  |

Table S2 Details on the capturing and monitoring of ten lynx in the Bohemian Forest Ecosystem used in this study. ^a^ at the beginning of lynx monitoring

| Individual | Sex | Monitoring | | | |
| --- | --- | --- | --- | --- | --- |
|  |  | Begin | Minimum age^a^ (years) | End | Number of captures |
| L1 | m | 15/01/2011 | 1 | 20/05/2012 | 1 |
| L2 | m | 22/03/2011 | 3 | 01/11/2012 | 1 |
| L3 | f | 17/03/2010 | 3 | 08/11/2011 | 2 |
| L4 | f | 17/03/2010 | <1 | 03/08/2012 | 2 |
| L5 | m | 07/03/2005 | 5 | 13/02/2010 | 3 |
| L6 | m | 11/03/2012 | 3 | 13/09/2013 | 1 |
| L7 | f | 17/01/2007 | 5 | 12/03/2008 | 1 |
| L8 | m | 18/02/2007 | 3 | 23/03/2011 | 2 |
| L9 | f | 27/02/2011 | 2 | 11/03/2012 | 1 |
| L10 | m | 27/03/2010 | 5 | 13/07/2010 | 1 |

Table S3 Summary of merged land-cover types and associated subcategories of the used land cover map based on expert judgements

| Land-cover type | Subcategories |
| --- | --- |
| Artificial | Railway |
|  | Residential area |
|  | Road |
| Clear-cut | Clear-cut area |
| Disturbance area | Dead wood - lying |
|  | Dead wood - lying - coniferous regeneration |
|  | Dead wood - lying - deciduous regeneration |
|  | Dead wood - lying - mixed regeneration |
|  | Dead wood - standing |
| Ecotone | Ecotone |
| Meadow | Meadow - cultivated |
|  | Meadow - natural |
|  | Meadow - wetland |
| Stand - mature | Coniferous stand - mature |
|  | Deciduous stand - mature |
|  | Mixed stand - mature |
| Stand - medium | Coniferous stand - medium |
|  | Deciduous stand - medium |
|  | Mixed stand - medium |
| Stand - young | Coniferous stand - young |
|  | Deciduous stand - young |
|  | Mixed stand - young |
| Others | Peat bog |
|  | Rock |
|  | Scrub pine |
|  | Water body |

Table S4 Use of forest types (based on tree species; %) by lynx in the Bohemian Forest Ecosystem during various phases of the day (daytime, night-time) and seasons (summer, winter)

|  | Day | | Night | |
| --- | --- | --- | --- | --- |
|  | Summer | Winter | Summer | Winter |
| Coniferous stand | 39.9 | 43.2 | 31.0 | 31.6 |
| Deciduous stand | 13.8 | 13.9 | 8.0 | 12.9 |
| Mixed stand | 20.4 | 28.9 | 16.7 | 26.2 |

Table S5 Summary of generalized additive mixed models predicting habitat selection by lynx in the Bohemian Forest Ecosystem based on tree species (in reference to the selection of coniferous stands (Intercept)). The estimates of the coefficients, standard errors (SE), z values and p-values (=Pr(>|z|)) are shown for these land-cover types

|  | Summer day | | | | Winter day | | | |
| --- | --- | --- | --- | --- | --- | --- | --- | --- |
| Variables | Esti-mate | SE | z value | Pr(>\|z\|) | Esti-mate | SE | z value | Pr(>\|z\|) |
| (Intercept) | -2.506 | 0.069 | -36.134 | <0.001 | -2.621 | 0.106 | -24.684 | <0.001 |
| Deciduous stand | 0.253 | 0.114 | 2.223 | 0.026 | 0.113 | 0.120 | 0.943 | 0.346 |
| Mixed stand | 0.035 | 0.096 | 0.365 | 0.715 | 0.165 | 0.093 | 1.777 | 0.076 |
|  | Summer night | | | | Winter night | | | |
| Variables | Esti-mate | SE | z value | Pr(>\|z\|) | Esti-mate | SE | z value | Pr(>\|z\|) |
| (Intercept) | -2.641 | 0.055 | -48.279 | <0.001 | -2.774 | 0.092 | -30.194 | <0.001 |
| Deciduous stand | -0.033 | 0.123 | -0.272 | 0.786 | 0.266 | 0.122 | 2.171 | 0.030 |
| Mixed stand | 0.121 | 0.095 | 1.273 | 0.203 | 0.350 | 0.097 | 3.621 | <0.001 |
